# A Review of Feature Extraction Software for Microarray Gene Expression Data

**DOI:** 10.1155/2014/213656

**Published:** 2014-08-31

**Authors:** Ching Siang Tan, Wai Soon Ting, Mohd Saberi Mohamad, Weng Howe Chan, Safaai Deris, Zuraini Ali Shah

**Affiliations:** Artificial Intelligence and Bioinformatics Research Group, Faculty of Computing, Universiti Teknologi Malaysia, 81310 Skudai, Johor, Malaysia

## Abstract

When gene expression data are too large to be processed, they are transformed into a reduced representation set of genes. Transforming large-scale gene expression data into a set of genes is called feature extraction. If the genes extracted are carefully chosen, this gene set can extract the relevant information from the large-scale gene expression data, allowing further analysis by using this reduced representation instead of the full size data. In this paper, we review numerous software applications that can be used for feature extraction. The software reviewed is mainly for Principal Component Analysis (PCA), Independent Component Analysis (ICA), Partial Least Squares (PLS), and Local Linear Embedding (LLE). A summary and sources of the software are provided in the last section for each feature extraction method.

## 1. Introduction

The advances of microarray technology allow the expression levels of thousands of genes to be measured simultaneously [1]. This technology has caused an explosion in the amount of microarray gene expression data. However, the gene expression data generated are high-dimensional, containing a huge number of genes and small number of samples. This is called the “large *p* small *n* problem” [2]. The high-dimensional data are the main problem when analysing the data. As a result, instead of using gene selection methods, feature extraction methods are also important in order to reduce the dimensionality of high-dimensional data. Instead of eliminating irrelevant genes, feature extraction methods work by transforming the original data into a new representation. Feature extraction is usually better than gene selection in terms of causing less information loss. As a result, the high-dimensionality problem can be solved using feature extraction.

Software is a set of machine readable instructions that direct a computer's processor to perform specific operations. With increases in the volume of data generated by modern biomedical studies, software is required to facilitate and ease the understanding of biological processes. Bioinformatics has emerged as a discipline in which emphasis is placed on easily understanding biological processes. Gheorghe and Mitrana [3] relate bioinformatics to computational biology and natural computing. Higgs and Attwood [4] believe that bioinformatics is important in the context of evolutionary biology.

In this paper, the software applications that can be used for feature extraction are reviewed. The software reviewed is mainly for Principal Component Analysis (PCA), Independent Component Analysis (ICA), Partial Least Squares (PLS), and Local Linear Embedding (LLE). In the last section for each feature extraction method, a summary and sources are provided.

## 2. Software for Principal Component Analysis (PCA)

In the domain of dimension reduction, PCA is one of the renowned techniques. The fundamental concept of PCA is to decrease the dimensionality of a given data set, whilst maintaining as plentiful as possible the variation existing in the initial predictor variables. This is attained by transforming the *p* initial variables *X* = [*x*_1_, *x*_2_,…, *x*_*p*_] to a latest set of *q* predictor variables. Linear amalgamation of the initial variables is *T* = [*t*_1_, *t*_2_,…, *t*_*q*_]. In mathematical domain, PCA successively optimizes the variance of a linear amalgamation of the initial predictor variables:

(1) $$\begin{array}{c} u_{q}=\text{argmax}\left(\mathrm{Var}\left(Xu\right)\right),\\ u^{t}u=1 \end{array}$$

conditional upon the constraint *u*_*i*_^*T*^ *S*_*X*_ *u*_*j*_ = 0, for every 1 ≤ *i* ≤ *j*. The orthogonal constraint makes sure that the linear combinations are uncorrelated; that is, Cov(*Xu*_*i*_, *Xu*_*j*_) = 0, *i* ≠ *j*. These linear combinations are denoted as the principle components (PCs):

(2) $$\begin{array}{c} t_{i}={Xu}_{i}. \end{array}$$

The projection vectors (or known as the weighting vectors) *u* can be attained by eigenvalue decomposition on the covariance matrix *S*_*X*_:

(3) $$\begin{array}{c} S_{X}u_{i}=\gamma_{i}u_{i}, \end{array}$$

where *γ*_*i*_ is the *i*th eigenvalue in the decreasing order, for *i* = 1,…, *q*, and *u*_*i*_ is the resultant eigenvector. The eigenvalue *γ*_*i*_ calculates the variance of the *i*th PC and the eigenvector *u*_*i*_ gives the weights for the linear transformation (projection).

### 2.1. FactoMineR

FactoMineR is an R package that provides various functions for the analysis of multivariate data [5]. The newest version of this package is maintained by Hussen et al. [6]. There are a few main features provided by this package; for example, different types of variables, data structures, and supplementary information can be taken into account. Besides that, it offers dimension reduction methods such as Principal Component Analysis (PCA), Multiple Correspondence Analysis (MCA), and Correspondence Analysis (CA). The steps in implementing PCA are described in Lê et al. [5] and Hoffmann [7]. For PCA, there are three main functions for performing the PCA, plotting it, and printing its results. This package is mainly for Windows, MacOS, and Linux.

### 2.2. ExPosition

ExPosition is an R package for the multivariate analysis of quantitative and qualitative data. ExPosition stands for Exploratory Analysis with the Singular Value Decomposition. The newest version of this package is maintained by Beaton et al. [8]. A variety of multivariate methods are provided in this package such as PCA, multidimensional scaling (MDS), and Generalized PCA. All of these methods can be performed by using the* corePCA* function in this package. Another function,* epPCA*, can be applied to implement PCA. Besides that, Generalized PCA can be implemented using the function* epGPCA* as well. All of these methods are used to analyse quantitative data. A plotting function is also offered by this package in order to plot the results of the analysis. This package can be installed on Windows, Linux, and MacOS.

### 2.3. amap

The R package “amap” was developed for clustering as well as PCA for both parallelized functions and robust methods. It is an R package for multidimensional analysis. The newest version is maintained by Lucas [9]. Three different types of PCA are provided by this package. The methods are PCA, Generalized PCA, and Robust PCA. PCA methods can be implemented using the functions* acp* and* pca* for PCA,* acpgen* for Generalized PCA, and* acprob* for Robust PCA. This package also allows the implementation of correspondence factorial analysis through the function* afc*. Besides that, a plotting function is also provided for plotting the results of PCA as a graphical representation. The clustering methods offered by this package are* k*-means and hierarchical clustering. The dissimilarity matrix and distance matrix can be computed using this package as well. This package is mainly for Windows, Linux, and MacOS.

### 2.4. ADE-4

ADE-4 was originally developed by Thioulouse et al. [10] as software for analyzing multivariate data and displaying graphics. This software includes a variety of methods such as PCA, CA, Principal Component Regression, PLS, Canonical Correspondence Analysis, Discriminant Analysis, and others. Besides that, this software is implemented in an R environment as an R package, “ade4.” The newest version of this package is maintained by Penel [37]. In this package, PCA can be performed by using the* dudi.pca* function. A visualization function is also provided in order to visualize the results as a graphical representation. In previous studies, this package was implemented by Dray and Dufour [38] to identify and understand ecological community structures. This package is mainly for Linux, Windows, and MacOS.

### 2.5. MADE4

MADE4 (microarray ade4) was developed by Culhane et al. [11] for multivariate analysis of gene expression data based on the R package “ade4.” Basically, it is the extensions of the R package “ade4” for microarray data. The purpose of writing this software was to help users in the analysis of microarray data using multivariate analysis methods. This software is able to handle a variety of gene expression data formats, and new visualization software has been added to the package in order to facilitate the visualization of microarray data. Other extra features such as data preprocessing and gene filtering are included as well. However, this package was further improved by the addition of the LLSimpute algorithm to handle the missing values in the microarray data by Moorthy et al. [39]. It is implemented in an R environment. The advance of this package is that multiple datasets can be integrated to carry out analysis of microarray data. The newest version is maintained by Culhane [40]. This package can be installed on Linux, Windows, and MacOS.

### 2.6. XLMiner

XLMiner is add-in software for Microsoft Excel that offers numerous data mining methods for analysing data [12]. It offers a quick start in the use of a variety of data mining methods for analysing data. This software can be used for data reduction using PCA, classification using Neural Networks or Decision Trees [41, 42], class prediction, data exploration, affinity analysis, and clustering. In this software, PCA can be implemented using the Principle Component tab [43]. This software is implemented in Excel. As a result, the dataset should be in an Excel spreadsheet. In order to start the implementation of XLMiner, the dataset needs to be manually partitioned into training, validation, and test sets. Please see http://www.solver.com/xlminer-data-mining for further details. This software can be installed on Windows and MacOS.

### 2.7. ViSta

ViSta stands for Visual Statistics System and can be used for multivariate data analysis and visualization in order to provide a better understanding of the data [13]. This software is based on the Lisp-Stat system [44]. It is an open source system that can be freely distributed for multivariate analysis and visualization. PCA and multiple and simple CA are provided in this software. Its main advance is that the data analysis is guided in a visualization environment in order to generate more reliable and accurate results. The four state-of-the-art visualization methods offered by this software are GuideMaps [45], WorkMaps [46], Dynamic Statistical Visualization [47], and Statistical Re-Vision [48]. The plug-ins for PCA can be downloaded from http://www.mdp.edu.ar/psicologia/vista/vista.htm. An example of implementation of the analysis using PCA can be viewed in Valero-Mora and Ledesma [49]. This software can be installed on Windows, Unix, and Macintosh.

### 2.8. imDEV

Interactive Modules for Data Exploration and Visualization (imDEV) [14] is an application of RExcel that integrates R and Excel for the analysis, visualization, and exploration of multivariate data. It is used in Microsoft Excel as add-ins by using an R package. Basically, it is implemented in Visual Basic and R. In this software, numerous dimension reduction methods are provided such as PCA, ICA, PLS regression, and Discriminant Analysis. Besides that, this software also offers clustering, imputing of missing values, feature selection, and data visualization. The 2 × 3 visualization methods are offered such as dendrograms, distribution plots, biplots, and correlation networks. This software is compatible with a few versions of Microsoft Excel such as Excel 2007 and 2010.

### 2.9. Statistics Toolbox

Statistical Toolbox offers a variety of algorithms and tools for data modelling and data analysis. Multivariate data analysis methods are offered by this toolbox. The methods include PCA, clustering, dimension reduction, factor analysis, visualization, and others. In the statistical toolbox of MATLAB, several PCA functions are provided for multivariate analysis, for example,* pcacov*,* princomp*, and* pcares* (MathWorks). Most of these functions are used for dimensional reduction.* pcacov* is used for covariance matrices,* princomp* for raw data matrices, and* pcares* for residuals from PCA. All of these functions are implemented in MATLAB.

### 2.10. Weka

Weka [16] is data mining software that provides a variety of machine learning algorithms. This software offers feature selection, data preprocessing, regression, classification, and clustering methods [50]. This software is implemented in a Java environment. PCA is used as a dimension reduction method in Weka to reduce the dimensionality of complex data through transformation. However, not all of the datasets are complete. Prabhume and Sathe [51] introduced a new filter PCA for Weka in order to solve the problem of incomplete datasets. It works by estimating the complete dataset from the incomplete dataset. This software is mainly for Windows, Linux, and MacOS.

### 2.11. NAG Library

In NAG Library, the function of PCA is provided as the g03aa routine [17] in both C and Fortran. This routine performs PCA on data matrices. This software was developed by the Numerical Algorithms Group. In the NAG Library, more than 1700 algorithms are offered for mathematical and statistical analysis. For PCA, it is suitable for multivariate methods, G03. Other methods provided are correlation analysis, wavelet transforms, and partial differential equations. Please refer to http://www.nag.com/numeric/MB/manual_22_1/pdf/G03/ g03aa.pdf for further details about the g03aaa routine. This software can be installed on Windows, Linux, MacOS, AIX, HP UX, and Solaris.

### 2.12. Case Study

In this section, we will discuss the implementation of coinertia analysis (CIA) to cross-platform visualization in* MADE4* and* ADE4* to perform multivariate analysis of microarray datasets. To demonstrate, PCA was applied on 4 childhood tumors (NB, BL-NHL, EWS, and RMS) from a microarray gene expression profiling study [52]. From these data, a subset (*khan$train*, 206 genes × 64 cases), each case's factor denoting the respective class (*khan$train* classes, length = 64), and a gene annotation's data frame are accessible in aforementioned dataset in MADE4:

 
*< library (made4)*
 
*< data (khan)*
 
*< dataset = khan$train*
 
*< fac = khan$train.classes*
 
*< geneSym = khan$annotation$Symbol*
 
*< results.coa <- ord (dataset, type = “coa”)*
 
*< par (mfrow = c (1, 2))*
 
*< plotarrays (results.coa, classvec = fac)*
 
*< plotgenes (results.coa, genelabels = geneSym).*

**Figure 1:**
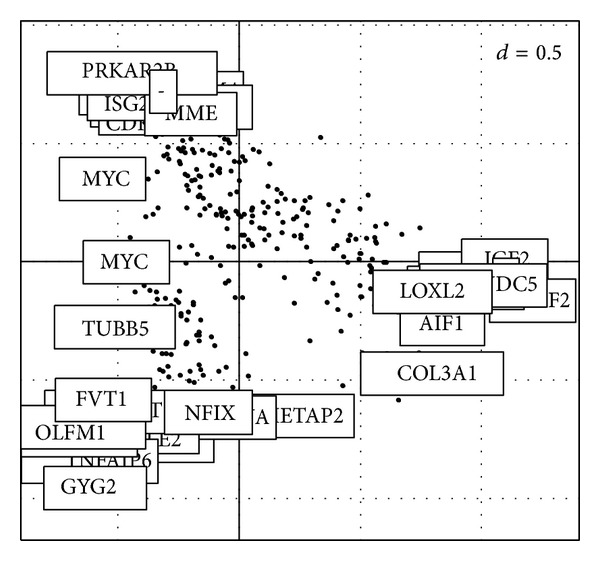
Plot of genes.

Figure 1 shows the PCA of a 306-gene subset. As origin as the point of reference, the more advanced gene and case are projected in the similar direction, the stronger the association between involved gene and case is (gene is upregulated in that array sample).

### 2.13. Summary of PCA Software

Tables 1 and 2 show the summary and sources of PCA software, respectively.  Table 3 discusses the related work of this software.

## 3. Software for Independent Component Analysis (ICA)

ICA is considered as a valuable extension of PCA that has been established considering the blind separation of independent sources from their linear combination [53]. In a way, the initial point of ICA is the property of uncorrelation of general PCA. Based on *n* × *p* data matrix *X*, whose rows *r*_*i*_  (*j* = 1,…, *n*) tally to observational variables and whose columns *c*_*j*_  (*j* = 1,…, *p*) are the individuals of the corresponding variables, the ICA model of *X* can be written as

(4) $$\begin{array}{c} X=AS. \end{array}$$

With generality intact, *A* is a *n* × *n* mixing matrix, whereas *S* is a *n* × *p* source matrix under the necessity of *S* being statistically independent as possible. “Independent components” are the new variables confined in the rows of *S*, to wit, the variables observed are linearly collected independent components. Mutual information *I* = ∑_*k*_ *H*(*S*_*k*_) − *H*(*S*), where *H*(*S*_*k*_) = −∫*p*(*S*_*k*_)log⁡⁡*p*(*S*_*k*_)*ds*_*k*_ is the marginal entropy of the variables *S*_*k*_, *p*(*S*_*k*_) is the probabilistic density function, and *H*(*S*) is the joint entropy [54]. Value the independent components able to be attained by discovering the correct linear mixtures of the observational variables, since mixing can be inverted as

(5) $$\begin{array}{c} U=S=A^{-1}X=WX. \end{array}$$

### 3.1. FastICA

FastICA is the most widely used method of ICA [55]. It is implemented in an R environment as the R package “FastICA” for performing ICA and Projection Pursuit by using the FastICA algorithm. FastICA was first introduced by Hyvärinen [54] for single and multiple component extraction. The FastICA algorithm is based on a fixed-point iteration scheme maximizing non-Gaussianity as a measure of statistical independence. This package is maintained by Marchini et al. [18]. ICA is used to extract the informative features through a transformation of the observed multidimensional random vectors into independent components. This package is mainly for Windows, Linux, and MacOS. FastICA is also implemented in MATLAB. In MATLAB, FastICA implements a fast fixed-point algorithm for ICA as well as projection pursuit. It provides a simple user interface and also a powerful algorithm for computation.

### 3.2. JADE

JADE is an R package that provides a function for implementing ICA. This package is maintained by Nordhausen et al. [19]. In this package, Cardoso's JADE algorithm [56] is provided for ICA. Instead of the JADE algorithm, other Blind Source Separation (BSS) methods such as the SOBI [57] and AMUSE [58] methods are offered. Both of these methods are mainly used for solving second order BSS problems. Amari error [59] is offered to evaluate the performance of the ICA algorithm. This package can be installed on Linux, Windows, and MacOS.

### 3.3. High Performance Signal Analysis Tools (HiPerSAT)

HiPerSAT is written in C++ for processing electroencephalography (EEG) data with whitening of data and ICA [20]. MPI and OpenMP are used to perform parallel analysis of ICA. Basically, this software is used to analyse EEG data in order to understand the neurological components of brain activity. In this software, FastICA, SOBI, and Informax algorithms are offered. HiPerSAT is integrated into MATLAB and EEGLAB [60]. EEGLAB is MATLAB-based software that is used for analysing EEG data. However, the advantage of HiPerSAT is that it can handle larger datasets compared to MATLAB. In comparison to EEGLAB, HiPerSAT is able to handle large datasets without partitioning but EEGLAB requires data partitioning. Data whitening is performed before implementing the algorithms. This software can be installed on all platforms.

### 3.4. MineICA

MineICA is an R package that supplies the implementation of ICA on transcriptomic data [21]. The main purpose of MineICA is to provide an easier way of interpreting the decomposition results from ICA. Besides that, this software also provides a correlation-based graph for comparing the components from different datasets. The newest version of this package is maintained by Biton [61]. This package provides some features such as storage of ICA results, annotation of features, and visualization of the results of ICA. This package can be installed on Linux, MacOS, and Windows.

### 3.5. Pearson Independent Component Analysis

Karnanen [22] developed an R package for a feature extraction technique based on the Pearson ICA algorithm. This is a mutual information-based blind source separation approach which applies the Pearson system as a parametric model. In order to extract the independent components using the ICA algorithm, the mutual information of the components has to be minimized. However minimization of mutual information is required to use a score function. The Pearson system was used to model the score function. The parameters of the Pearson system are estimated by the method of moments. In order to speed up the algorithm, tanh nonlinearity is used when the distribution is far from Gaussian.

### 3.6. Maximum Likelihood Independent Component Analysis

Teschenforff [23] developed an R package for ICA by using maximum likelihood estimation. This method was first introduced by Hyvaerinen et al. [62]. This method uses a fixed-point algorithm as the Maximum Likelihood estimation. For a fixed set of data and underlying statistical model, Maximum Likelihood selects the set of values of the model parameters that maximizes the likelihood function. Maximum Likelihood estimation gives a unified approach to estimation, which is well-defined in the case of normal distribution. By using a maximum likelihood framework and controlling the number of algorithm runs, this fixed-point algorithm provides a very fast implementation for maximization of likelihood.

### 3.7. Sample Case Study

In this section, we utilize* MineICA* for microarray-based gene expression data of 200 breast cancer tumors kept in the package* breastCancerMAINZ* [63] based on a study done by Biton et al. [21]. In this study, we focused on how* MineICA* can be utilized to study an ICA-based decomposition. Pseudo code for this case study is as follows:

1. Loading the library and the data
2. Creation of an* IcaSet* object
  (2.1) Load an example of expression data
  (2.2) Run ICA
  (2.3) Create a* MineICAParams* object, function* buildMineICAParams*
  (2.4) Create an* IcaSet* instance, function* buildIcaSet*
  (2.5) *IcaSet* basics
3. Run global analysis
4. Run analysis by calling individual functions
  (4.1) Write description of contributing genes or features, function* writeProjByComp*
  (4.2) Plot heatmaps of the contributing elements, function* plot_heatmapsOnSel*
  (4.3) Gene enrichment analysis, function* runEnrich*
  (4.4) Association with sample variables
  (4.5) Clustering of the samples according to each component
  (4.6) Comparison of* IcaSet* objects, function* runCompareIcaSets.*

**Figure 2:**
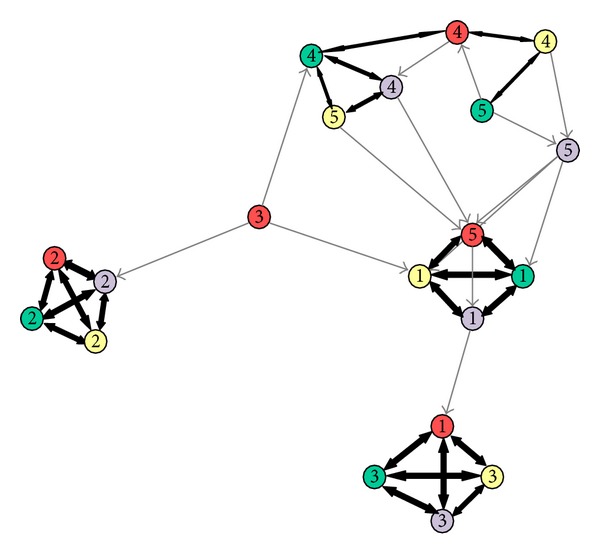
Correlation-based graph.

Figure 2 explains the correlation based graph denoting relationship between independent components (IC) attained on four breast cancer samples' microarray data. Every node represents an IC and respective colors denote the origin of dataset. Thickness of edge represents the extent of correlation among the linked ICs. Black edges represent reciprocal nodes.

### 3.8. Summary of ICA Software

Tables 4 and 5 show the summary and sources of ICA software, respectively.

## 4. Software for Partial Least Squares (PLS)

The fundamental hypothesis of PLS is that the experimental information is created by a framework or methodology which is determined by a small number of latent characteristics. Thusly, PLS goes for discovering uncorrelated linear transformation of the initial indicator characteristics which have high covariance with the reaction characteristics. In light of these latent components, PLS predicts reaction characteristics *y*, the assignment of regression, and reproduce initial matrix *X*, the undertaking of data modelling, in the meantime. The purpose of building components in PS is to optimize the covariance among the variable *y* and the initial predictor variables *X*:

(6) $$\begin{array}{c} w_{q}=\text{argmax}\left(\text{Cov}\left(X_{w},y\right)\right),\\ w^{T}w=1. \end{array}$$

Restricted to constraint *w*_*i*_^*T*^ *S*_*x*_ *w*_*j*_ = 0, for all 1 ≤ *i* < *j*. The crucial assignment of PLS is to attain the vectors of maximum weights *w*_*i*_  (*i* = 1,…, *q*) to build a small number of components, while PCA is an “unsupervised” method that utilizes the *X* data only. To develop the components, [*t*_1_, *t*_2_,…, *t*_*q*_], PLS decomposes *X* and *y* to yield a bilinear denotation of the data [64]:

(7) $$\begin{array}{c} X=t_{1}w_{1}^{T}+t_{2}w_{2}^{T}+\cdots +t_{q}w_{q}^{T}+e,\\ y=t_{1}v_{1}^{T}+t_{2}v_{2}^{T}+\cdots +t_{K}v_{q}^{T}+f, \end{array}$$

where *w*'s are vectors of weights for building the PLS components *t* = *X*_*w*_, *v*'s are scalars, and *e* and *f* are the residuals. The concept of PLS is to assume *w* and *v* by regression.

### 4.1. Partial Least Squares Discriminant Analysis

Barker and Rayens [24] developed a PLS for discriminant analysis. However the original PLS was not designed for discriminant purposes. PLS Discriminant Analysis is used to find a linear regression model by projecting the dependent features and the independent features to a new space. Then the fundamental relations can be extracted from the latent variables. This method was developed for software called Unscrambler, which was first developed by Martens and Naes [65]. Unscrambler is a commercial software product for multivariate data analysis. Unscrambler is used for analysing large and complex datasets quickly and easily using the power of multivariate analysis. Moreover this multivariate data analysis also offers exceptional data visualization.

### 4.2. Least Squares: Partial Least Squares

Jørgensen et al. [25] proposed a method of using an iterative combination of PLS and ordinary least squares to extract the relationship between the predictor variable and the responses. This method is based on a combination of least squares estimates for the design variables and PLS regression on the spectra. The PLS scores were incorporated into the ordinary least squares equation on the spectra. The idea is to separate the information from the spectral and design matrices in a nice way. However this method is able to extract the information even when fewer components are used. In addition, this method is insensitive to the relative scaling of the spectra and the process. Moreover this combination method is also less biased than the individual PLS technique.

### 4.3. Powered Partial Least Squares Discriminant Analysis

Liland and Indahl [26] extended the Powered PLS to Powered PLS Discriminant Analysis to overcome the extraction of information for the multivariate classification problem. This method can construct more efficient group separation and generate more interpretive outcomes than the ordinary Partial Least Square Discriminant Analysis technique. The features extracted by the Powered PLS can contribute to revealing the relevance of particular predictors and often requires smaller and simpler components than ordinary PLS. Moreover the optimization task is equivalent to maximizing the correlation between the transformed predictors and the groups. This makes it possible to discard the influence of less important predictors. This method was also developed by the authors for availability in an R package.

### 4.4. Penalized Partial Least Squares

Krämer et al. [27] proposed a combination of the feature extraction technique PLS with a penalization framework. This method is an extension of PLS regression using a penalization technique. Ordinary PLS is suited for regression problems by minimizing a quadratic loss function iteratively. In addition, the representation in terms of kernel matrices provides an intuitive geometric interpretation of the penalty term. The penalty terms control the roughness of the estimated functions. With the incorporation of penalization into this framework, the research direction became more promising. This method is used to extract relevant information for high-dimensional regression problems and also for noisy data. This method was also developed by the Krämer and her colleagues colleagues [66] for availability in an R package.

### 4.5. SlimPLS

Gutkin et al. [33] proposed a feature extraction method based on PLS called SlimPLS. Ranking-based filters usually utilize a univariate method when selecting features. The filter methods can produce reasonable feature sets especially when the original feature sets are uncorrelated. However the chosen feature set will be suboptimal when the features of the original set are dependent. Some of the features will add little discriminative power on top of previously selected features. SlimPLS is a multivariate feature extraction method which incorporates feature dependencies into calculation. This multivariate property is constructed by combining the highly predictive feature with some less predictive but correlated features. This is because the added features will provide more information on the behaviour of the samples.

### 4.6. Sparse Partial Least Squares Discriminant Analysis and Sparse Generalized Partial Least Squares

Chung and Keles [28] proposed two extension feature extraction approaches based on Sparse PLS. These approaches are Sparse PLS Discriminant Analysis and Sparse Generalized PLS for high-dimensional datasets. These two approaches improved ordinary PLS by employing feature extraction and dimension reduction simultaneously. These two approaches perform well even with unbalanced sample sizes of the classes. Sparse PLS Discrimination Analysis is computationally efficient because it only requires computational time for one run of Sparse PLS and a classifier. Moreover, Sparse Generalized PLS extends Sparse PLS to the generalized linear model framework. These methods were also developed by Chung and Keles for availability in an R package.

### 4.7. Degrees of Freedom of Partial Least Squares

Kramer and Sugiyama [29] proposed a method of unbiased estimation of the degrees of freedom for PLS regression. The authors stated that the construction of latent components from the independent variable also depended on the dependent variable. However for PLS regression, the optimal number of components needs to be determined first. One of the ways of determining the optimal number of components is through the degrees of freedom for the complexity of fitted models. Moreover the degrees of freedom estimate can be used for the comparison of different regression methods. Furthermore, the two implementations for the degrees of freedom utilize the connection between PLS regression and numerical linear methods from numerical linear. The authors also developed an R package for this unbiased estimation of the degrees of freedom of PLS.

### 4.8. Surrogate Variable Analysis Partial Least Squares

Chakraborty and Datta [30] proposed a surrogate variable analysis method based on PLS. In differential gene expression analysis, one of the important issues is to avoid the hidden confounders in the dataset. The hidden confounders of gene expression are caused by different environmental conditions of the samples. However this problem cannot be simply overcome by modifying the gene expression data by using a normalizing technique. This method can extract the informative features by identifying the hidden effects of the underlying latent factors using ordinary PLS and applying analysis of covariance (ANCOVA). ANCOVA is applied with the PLS signatures of these hidden effects as covariates in order to identify the genes that are truly differentially expressed. This method was also developed by the authors for availability in an R package.

### 4.9. Partial Least Squares Path Modelling

Sanchez and Trinchera [31] developed an R package for Partial Least Squares Path Modelling (PLS-PM). PLS-PM was first introduced by Wold [67] and is also known as Structural Equation Modelling (SEM). It can be used as a composite-based alternative to factor-based SEM. PLS-PM can be used when the distributions are highly skewed. Moreover, PLS-PM can also be used to estimate relationships between latent variables with several indicators even though the sample size is small. Basically, PLS-PM consists of two sets of linear equations: the inner model and the outer model. The inner model specifies the relations between latent variables, while the outer model specifies the relations between a latent variable and its observed indicator. PLS-PM is a multivariate feature extraction analysis technique based on the cause-effect relationships of the unobserved and observed features.

### 4.10. Partial Least Squares Regression for Generalized Linear Models

Bertrand et al. [32] developed a software application of PLS regression for generalized linear models. Generalized linear models are important to allow the response features to have a distribution other than normal. Generalized linear models can be viewed as a case of generalized linear models with an identity link. From the perspective of generalized linear models, however, it is useful to suppose that the distribution function is the normal distribution with constant variance and the link function is the identity, which is the canonical link if the variance is known. However, the generalized linear models preserve all the predictive power of the features where the predicted means are not assumed to be normally distributed. PLS regression is used to extract the predictive features from the generalized linear models.

### 4.11. Case Study

In this section, we will discuss the R package consists of svpls. This function will call fitModel function in order to appropriate a number of ANCOVA models that are identified by pmax to the data and opt for the best model by looking the minimum value of the Akaike's information Criterion (AIC) [68]. Subsequently, this model is utilized to forecast the real pattern of genes' differential expression. The command lines in R are as follows:

 > ## Fitting the optimal ANCOVA model to the data gives:
 > fit <-svpls (10, 10, hidden_fac.dat, pmax = 5, fdr = 0.05)
 > ## The optimal ANCOVA model, its AIC value and the positive genes detected
 > ## from it are givenL
 > fit$opt.model [1]
 > fit$AIC.opt [1]
 > fit$genes
 > ## The corrected gene expression matrix obtained after removing the effects of the hidden variability is given by:
 > Y.corrected <- fit$Y.corr
 > pval.adj <-fit$pvalues.adj.

**Figure 3:**
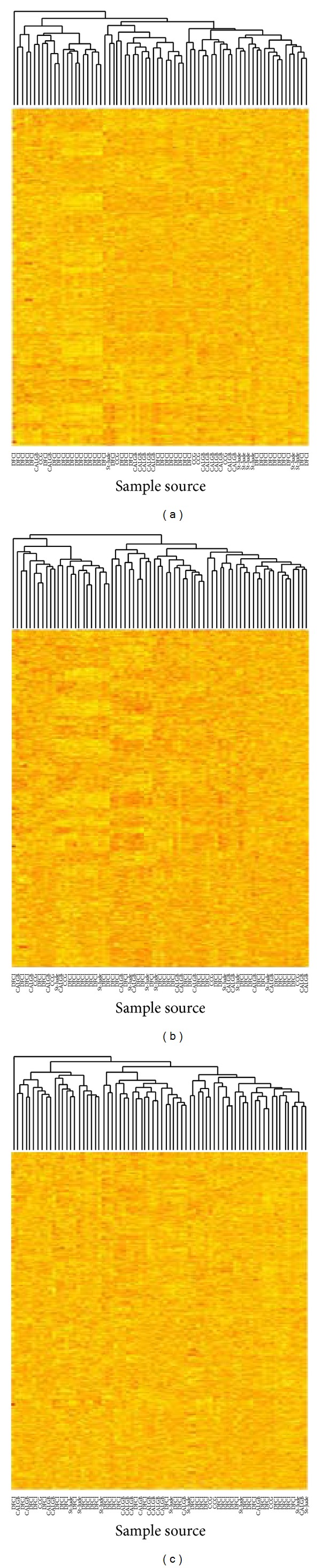
(a, b, and c) Heatmaps showing the original and corrected expression levels for the first 1000 genes in the Golub data. (a) Heatmap for the first 1000 genes in the original Golub expression data. (b) Heatmap for the first 1000 genes in the adjusted Golub expression data obtained by use of the R package ber. (c) Heatmap for the first 1000 genes in the adjusted Golub expression data obtained by the use of our R package svapls.

For instance, we study the efficacy of svapls on ALL/AML preprocessed dataset [69]. This data consists of expression levels of 7129 genes that have been log-transformed over two samples of patients. These two sets of 47 patients and 25 patients reported to suffer from Acute lymphoblastic Leukemia (ALL) and Acute Myeloid Leukemia (AML), respectively. By using svpls function, we yielded initial 1000 genes with corrected expression matrix. Random samples' distribution from four sources in the abovementioned matrix removes the extra effects owing to reported batch specific clustering in the initial data. In this context svapls performed equally efficient relative to another popular R package ber for removing batch effects in microarray data as shown in Figure 3.

### 4.12. Summary of PLS Software

Tables 6 and 7 show the summary and sources of PLS software, respectively.  Table 8 shows the related works on discussed software.

## 5. Software for Local Linear Embedding (LLE)

Straightforward geometric intuitions are the basis for LLE algorithm. Assume that given data comprise of *N* real-valued vectors *X*_*i*_, for each *D* dimensionality, tested by some core manifold. Given that there is adequate data, every data point and their neighbors are expected to be situated on or near to a locally linear patch of the manifold. Abovementioned patches are described by linear coefficients that rebuild every data point from respective neighbors. Equation (8) is the cost function used to calculate reconstruction errors which sums the squared distances between all the data points and their reconstructions. The weights *W*_*ij*_ summarize the contribution of the *j*th data point to the *i*th reconstruction. The optimal weights *W*_*ij*_ are found by solving a least-squares problem [70]:

(8) $$\begin{array}{c} \epsilon \left(W\right)=\overset{N}{\underset{i=1}{\sum }}{\left|X_{i}-\overset{K}{\underset{j=1}{\sum }}W_{ij}X_{j}\right|}^{2}=\underset{i=1}{\sum }\epsilon^{i}\left(W\right), \end{array}$$

(9) $$\begin{array}{c} \epsilon^{i}\left(W\right)={\left|\overset{K}{\underset{j=1}{\sum }}W_{j}^{i}\left(x_{i}-x_{j}\right)\right|}^{2}=\overset{k}{\underset{j=1}{\sum }}\;\overset{k}{\underset{m=1}{\sum }}W_{j}^{i}W_{m}^{i}Q_{jm}^{i}, \end{array}$$

(10) $$\begin{array}{c} Q_{jm}^{i}={\left(x_{i}-x_{j}\right)}^{T}\left(x_{i}-x_{m}\right)=\frac{\left(D_{i,j}+D_{i,m}-D_{j,m}\right)}{2}. \end{array}$$

### 5.1. lle

An R package “lle” has been developed in order to implement LLE for feature extraction. This package provides the algorithm of LLE in order to transform high-dimensional data into low-dimensional data. The newest version of this package is maintained by Diedrich and Abel [34]. The main functions of this package allow users to perform LLE and also to plot the results of LLE. The implementation of LLE is based on the idea of Ridder and Duin [71]. Besides that, some enhancements such as selection of the subset and calculation of the intrinsic dimension are offered. This package can be installed on Windows, Linux, and MacOS.

### 5.2. RDRToolbox

RDRToolbox is an R package developed for nonlinear dimension reduction with LLE and Isomap. The package is maintained by Bartenhagen [35]. It offers the transformation of high-dimensional to low-dimensional data by using either LLE or Isomap. Besides that, a plotting function is provided to plot the results. In addition, the Davis-Bouldin Index is provided for the purposes of validating clusters. It is mainly for Linux, MacOS, and Windows.

### 5.3. Scikit-Learn

Scikit-learn is software implemented in Python by integrating machine learning algorithms [36]. It is a simple-to-use software that allows users to implement a variety of machine learning algorithms. The machine learning algorithms include classification, clustering, feature extraction, model selection, manifold learning, and other methods. Isomap, LLE, and Local Tangent Space Alignment (LTSA) are provided by this software. Please see http://scikit-learn.org/stable/ for further details. This software can be installed on a variety of platforms such as Windows and Ubuntu.

### 5.4. Case Study

This section demonstrates the dimension reduction workflow for the publicly available the Golub et al. leukemia dataset (see Figure 5). The data is available as R package and can be downloaded and loaded via

 > source (“http://bioconductor.org/biocLite.R”)
 > biocLite (“golubEsets”)
 > library (golubEsets)
 > data (Golub_Merge).

The dataset consists of 72 samples, divided into 47 ALL and 25 AML patients, and 7129 expression values. In this example, we compute a two-dimensional LLE and Isomap embedding and plot the results. At first, we extract the features and class labels:

 > golubExprs = t (exprs (Golub_Merge))
 > labels = pData (Golub_Merge)$ALL.AML
 > dim (golubExprs).

The residual variance of Isomap can be used to estimate the intrinsic dimension of the dataset:

 > Isomap (data = golubExprs, dims = 1 : 10, plotResiduals = TRUE, *k* = 5).

**Figure 4:**
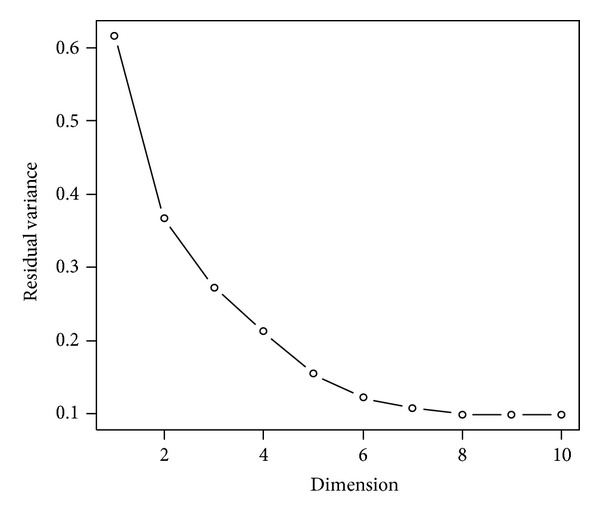
Plot of dimension versus residual variance.

**Figure 5:**
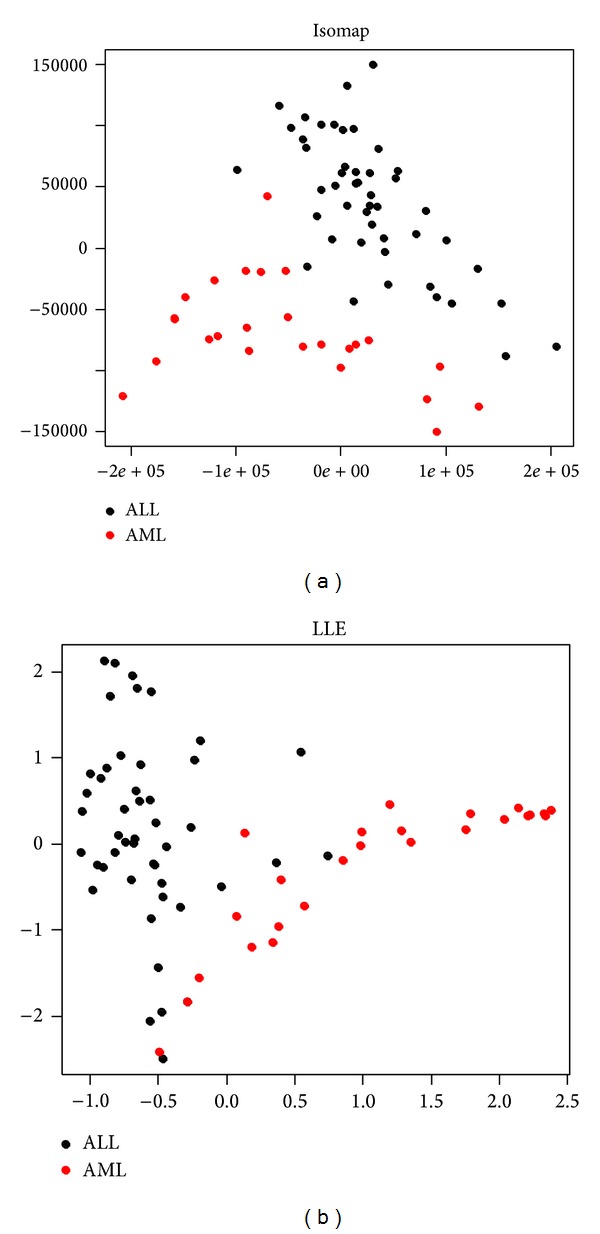
Two-dimensional embedding of the Golub et al. [69] leukemia dataset (top: Isomap; bottom: LLE).

Based on Figure 4, regarding the dimensions for which the residual variances stop to decrease significantly, we can expect a low intrinsic dimension of two or three and, therefore, visualization true to the structure of the original data. Next, we compute the LLE and Isomap embedding for two target dimensions:

 > golubIsomap = Isomap (data = golubExprs, dims = 2, *k* = 5)
 > golubLLE = LLE(data = golubExprs, dim = 2, *k* = 5).

The Davis-Bouldin-Index shows that the ALL and AML patients are well separated into two clusters:

 > DBIndex(data = golubIsomap$dim2, labels = labels)
 > DBIndex(data = golubLLE, labels = labels).

Finally, we use plotDR to plot the two-dimensional data:

 > plotDR(data = golubIsomap$dim2, labels = labels, axesLabels = c(“”, “”), legend = TRUE)
 > title (main = “Isomap”)
 > plotDR (data = golubLLE, labels = labels, axesLabels = c(“”,“”), legend = TRUE)
 > title (main = “LLE”).

Both visualizations, using either Isomap or LLE, show distinct clusters of ALL and AML patients, although the cluster overlaps less in the Isomap embedding. This is consistent with the DB-Index, which is very low for both methods, but slightly higher for LLE. A three-dimensional visualization can be generated in the same manner and is best analyzed interactively within R.

### 5.5. Summary of LLE Software

Tables 9 and 10 show the summary and sources of LLE software, respectively.  Table 11 shows the related works in discussed software.

## 6. Conclusion

Nowadays, numerous software applications have been developed to help users implement feature extraction of gene expression data. In this paper, we present a comprehensive review of software for feature extraction methods. The methods are PCA, ICA, PLS, and LLE. These software applications have some limitations in terms of statistical aspects as well as computational performance. In conclusion, there is a need for the development of better software.

## Figures and Tables

**Table 1:** A summary for PCA software.

Number	Software	Author/year	Language	Features
1	FactoMineR	Lê et al. [5]	R	(i) Various dimension reduction methods such as PCA, CA, and MCA (ii) Different types of variables, data structures, and supplementary information are considered (iii) The PCA function can handle missing values

2	ExPosition	Beaton et al. [8]	R	(i) Numerous multivariate analysis methods such as PCA and Generalized Principal Component Analysis (GPCA) (ii) Can be applied to quantitative and qualitative data (iii) Implementation of Singular Value Decomposition

3	amap	Lucas [9]	R	(i) Different types of PCA are provided: PCA, Generalized PCA, and Robust PCA (ii) Clustering methods are provided such as hierarchical clustering and *k*-means clustering (iii) Plotting function for PCA (iv) Computing distance and dissimilarity matrices

4	ADE-4	Thioulouse et al. [10]	R	A variety of methods such as PCA, CA, Principal Analysis Regression, PLS, and others are offered

5	MADE4	Culhane et al. [11]	R	(i) Functions provided by ADE-4 (ii) Integration of multiple datasets for multivariate analysis (iii) Functions for visualizing and plotting the results of analysis, including 3D plots (iv) Addition of LLSimpute algorithm for imputation of missing values

6	XLMiner	Witten and Frank [12]	Implemented in Excel	(i) Provision of data reduction methods such as PCA (ii) Can be used for classification, clustering, data preprocessing, data normalization, and others

7	ViSta	Young et al. [13]	C++, Fortran, XLisp, and ViDAL	(i) Multivariate analysis methods are offered such as PCA, Interactive Cluster Analysis, and Parallel Boxplots (ii) Provision of dynamic and high-interaction visualization for displaying multiple views of data

8	imDEV	Grapov and Newman [14]	Visual Basic and R	(i) Data preprocessing: missing values imputation and data transformations (ii) Clustering methods are offered (iii) Dimension reduction methods: PCA and ICA (iv) Feature selection methods (v) Visualization of data dependencies

9	Statistics Toolbox	The MathWorks [15]	MATLAB	(i) Multivariate statistics such as PCA, clustering, and others (ii) Statistical plots, probability distributions, linear models, nonlinear models for regression, and others are provided

10	Weka	Hall et al. [16]	Java	A variety of machine learning algorithms are provided such as feature selection, data preprocessing, regression, dimension reduction, classification, and clustering methods

11	NAG Library	NAG Toolbox for MATLAB [17]	Fortran and C	(i) Provision of more than 1700 mathematical and statistical algorithms (ii) Multivariate analysis using PCA can be implemented using the g03aa routine

**Table 2:** Sources of PCA software.

Number	Software	Sources
1	FactoMineR	http://cran.r-project.org/web/packages/FactoMineR/index.html
2	ExPosition	http://cran.r-project.org/web/packages/ExPosition/index.html
3	Amap	http://cran.r-project.org/web/packages/amap/index.html
4	ADE-4	http://cran.r-project.org/web/packages/ade4/index.html
5	MADE4	http://www.bioconductor.org/packages/2.11/bioc/html/made4.html
6	XLMiner	http://www.solver.com/xlminer-data-mining
7	ViSta	http://www.visualstats.org/ http://www.mdp.edu.ar/psicologia/vista/vista.htm
8	imDEV	http://sourceforge.net/projects/imdev/
9	Statistics Toolbox	http://www.mathworks.com/matlabcentral/fileexchange/30792-pca-principal-component-analysis
10	Weka	http://www.cs.waikato.ac.nz/ml/weka/downloading.html
11	NAG Library	http://www.nag.com/downloads/cldownloads.asp

**Table 3:** Related work.

Software	Author	Motivation	Advantage
FactoMineR	Lê et al. (2009) [5]	(i) Providing a multivariate data analytic technique for applications in biological systems (ii) To combine “Omics” data structured into groups (iii) To help on their functional interpretations.	(i) It provides a geometrical point of view and a lot of graphical outputs (ii) It can take into account a structure on the data (iii) A GUI is available.

MADE4	Culhane et al. [11]	To provide a simple-to-use tool for multivariate analysis of microarray data	(i) Accepts a wide variety of gene-expression data input formats (ii) No additional data processing is required

Statistic toolbox	The MathWorks [15]	High-dimensional and complex microarray data need automatic/computer aided tools for analysis	Elegant matrix support; visualization

imDev	Grapov and Newman, 2012 [14]	Omics experiments generate complex high-dimensional data requiring multivariate analyses	(i) User-friendly graphical interface (ii) Visualizations can be exported directly from the R plotting interface in a variety of file formats (iii) Dynamic loading of R objects between analyses sessions

**Table 4:** Summary of ICA software.

Number	Software	Author/year	Language	Features
1	FastICA	Marchini et al. [18]	R and MATLAB	ICA algorithm is provided for implementing the analysis using ICA

2	JADE	Nordhausen et al. [19]	R	(i) JADE algorithm is provided for ICA (ii) Other BSS methods such as AMUSE and SOBI are offered

3	HiPerSAT	Keith et al. [20]	C++, MATLAB, and EEGLAB	(i) Integration of FastICA, Informax, and SOBI algorithms (ii) Data whitening is provided

4	MineICA	Biton et al. [21]	R	(i) Storage and visualization of ICA results (ii) Annotation of features

5	Pearson ICA	Karnanen [22]	R	Extraction of the independent components using the minimization of mutual information from the Pearson system

6	Maximum Likelihood ICA	Teschenforff [23]	R	Implementation of the Maximum Likelihood and fixed-point algorithm into ICA

**Table 5:** Sources of ICA software.

Number	Software	Sources
1	FastICA	R: http://cran.r-project.org/web/packages/fastICA/index.html
		MATLAB: http://research.ics.aalto.fi/ica/fastica/
2	JADE	http://cran.r-project.org/web/packages/JADE/index.html
3	HiPerSAT	http://nic.uoregon.edu/projects/hipersat/index.php
4	MineICA	http://www.bioconductor.org/packages/2.12/bioc/html/MineICA.html
5	Pearson ICA	http://cran.r-project.org/web/packages/PearsonICA/index.html
6	Maximum Likelihood ICA	http://cran.r-project.org/web/packages/mlica2/index.html

**Table 6:** A summary of PLS software.

Number	Software	Author/year	Language	Features
1	PLS Discriminant Analysis	Barker and Rayens [24]	C/C++, Visual Basic	PLS for discriminant analysis

2	Least Squares–PLS	Jørgensen et al. [25]	R	Implementation combining PLS and ordinary least squares

3	Powered PLS Discriminant Analysis	Liland and Indahl [26]	R	Extraction of information for multivariate classification problems

4	Penalized PLS	Kr$\ddot{\text{a}}$mer et al. (2008) [27]	R	Extension of PLS regression using penalization technique

5	SlimPLS	Gutkin et al. [22]	R	Multivariate feature extraction method which incorporates feature dependencies

6	Sparse PLS Discriminant Analysis, Sparse Generalized PLS	Chung and Keles [28]	R	Sparse version techniques employing feature extraction and dimension reduction simultaneously

7	PLS Degrees of Freedom	Kramer and Sugiyama [29]	R	Using an unbiased estimation of the degrees of freedom for PLS regression

8	Surrogate Variable Analysis PLS	Chakraborty and Datta [30]	R	Extraction of the informative features with hidden confounders which are unaccounted for

9	PLS Path Modelling	Sanchez and Trinchera [31]	R	A multivariate feature extraction analysis technique based on the cause-effect relationships of the unobserved and observed features

10	PLS Regression for Generalized Linear Models	Bertrand et al. (2013) [32]	R	PLS regression is used to extract the predictive features from the generalized linear models

**Table 7:** Sources of PLS software.

Number	Software	Sources
1	PLS Discriminant Analysis	http://www.camo.com/downloads/sample-data.html
2	Least Squares–PLS	http://cran.r-project.org/web/packages/lspls/index.html
3	Powered PLS Discriminant Analysis	http://cran.r-project.org/web/packages/pls/index.html
4	Penalized PLS	http://cran.r-project.org/web/packages/ppls/index.html
5	SlimPLS	http://cran.r-project.org/web/packages/SlimPLS/index.html
6	Sparse PLS Discriminant Analysis, Sparse Generalized PLS	http://cran.r-project.org/web/packages/spls/index.html
7	Degrees of Freedom of PLS	http://cran.r-project.org/web/packages/plsdof/index.html
8	Surrogate Variable Analysis PLS	http://cran.r-project.org/web/packages/svapls/index.html
9	PLS Path Modelling	http://cran.r-project.org/web/packages/plspm/index.html
10	PLS Regression for Generalized Linear Models	http://cran.r-project.org/web/packages/plsRglm/index.html

**Table 8:** Related work.

Software	Author	Motivation	Advantage
plsRglm (R package)	Bertrand et al. (2010) [32]	(i) To deal with incomplete datasets using cross-validation (ii) To extend PLS regression to generalized linear models	(i) Provides formula support (ii) Several new classes and their generics (iii) Custom GLR models and graphics to assess the bootstrap based significance of the predictors

SVA-PLS	Chakraborty and Datta [30]	(i) To identify the genes that are differentially expressed between the samples from two different tissue types (ii) To identify the hidden effects of the underlying latent factors in a gene expression profiling study	(i) Relatively better at discovering a higher proportion of the truly significant genes (ii) Low error rate (iii) High sensitivity and specificity

SlimPLS	Gutkin et al. [33]	To obtain a low dimensional approximation of a matrix that is “as close as possible” to a given vector	(i) Focuses solely on feature selection (ii) Can be used as a pre-processing stage with different classifiers

**Table 9:** A summary of LLE software.

Number	Software	Author/year	Language	Features
1	lle	Diedrich and Abel [34]	R	(i) LLE algorithm is provided for transforming high-dimensional data into low-dimensional data (ii) Selection of subset and calculation of the intrinsic dimension are provided

2	RDRToolbox	Bartenhagen [35]	R	(i) LLE and Isomap for feature extraction (ii) Davis-Bouldin Index for the purpose of validating clusters

3	Scikit-learn	Pedregosa et al. [36]	Python	(i) Classification, manifold learning, feature extraction, clustering, and other methods are offered (ii) LLE, Isomap, and LTSA are provided

**Table 10:** Sources of LLE software.

Number	Software	Sources
1	lle	http://cran.r-project.org/web/packages/lle/index.html
2	RDRToolbox	http://www.bioconductor.org/packages/2.12/bioc/html/RDRToolbox.html
3	Scikit-learn	http://scikit-learn.org/dev/install.html

**Table 11:** Related work.

Software	Author	Motivation	Advantage
RDRToolbox	Bartenhagen [35]	(i) To reduce high dimensionality microarray data (ii) To preserve most of the significant information and generate data with similar characteristics like the high-dimensional original	(i) Combine information from all features (ii) Suited for low-dimensional representations of the whole data

Scikit-learn	Pedregosa et al. [36]	To calculate activity index parameters through clustering	(i) Easy-to-use interface (ii) Can easily be integrated into applications outside the traditional range of statistical data analysis

lle	Diedrich and Abel [34]	Currently available data dimension reduction methods are either supervised, where data need to be labeled, or computational complex	(i) Fast (ii) Purely unsupervised
